# Pain, Critical Care and Anesthesia section

**DOI:** 10.1186/s12967-014-0268-0

**Published:** 2014-10-08

**Authors:** Yasin Said AlMakadma

**Affiliations:** Sidra Medical and Research Center, Doha, Qatar

## Editorial

As we are witnessing major technological and informatics progress on a daily basis, the emphasis on translational approach in medical research has become not only clearly needed but also practically achievable. In addition, very closely linked fields such as Anesthesia, Pain Medicine and Critical Care may benefit mostly of the development and consolidation of translational medicine. From its “bench to bedside” and beyond, in the community, translational medicine touches the real goal of research which is to understand in order to correctly and adequately apply. Changes in the way we provide patients’ medical care require rigorous process and thorough analytical work so to reach the stage of implementing policies and strategies on the wider population.

Acute Respiratory Distress Syndrome (ARDS) is an example of how focus on translational research may lead to a better understanding of the disease. Defining ARDS continues to remain a challenge but it is also an essential step before including patients in ARDS related clinical trials. The clinical definition of ARDS and its diagnostic criteria have been subject to few revisions since the disease was initially described, in 1967, by Ashbaugh DG et al [1]. In 1994 the American European Consensus Criteria were used to redefine the disease [2]. However, ARDS was more recently reviewed again by the definition of the Berlin Consensus [3,4]. Despite of all these attempts, it remains true, however, that up to 50% of patients included in ARDS-related randomized trials may well be false positives. This would explain why over 200 randomized clinical trials had failed to identify consistent conclusions on therapeutic interventions as explained in the editorial of S. Fröhlich et al. who conclude that without specific biomarker, it is unlikely to make real progress in tackling this deadly disease [5]. On the other hand understanding and treating pain syndromes, such as Complex Regional Pain Syndrome (CRPS), still long way far from done. CRPS diagnosis relies mainly on bedside criteria and subjective findings and descriptions by both the patient and the physician [6,7]. Whether we consider “sensory sympathetic coupling”, “cortical re-organization” related to CRPS, underlying local mechanisms and genes translation in familial “CRPS” cases remain all to apprehend. Findings of Functional MRI are an example of how translational approach may well be of great value. Focused translational research may thus help us identifying biomarker(s) for the disease so that we may be able to start its management earlier and perhaps have less disabling outcome.

The concept of “Translational Medicine” represents a significant milestone in our journey towards better knowing and better managing diseases and towards better understanding how specific molecules, such as anaesthetics, anti-neuropathic and neuromodulation techniques such as Spinal Cord Stimulation affect the nervous system among others. This is why we are launching this section, Pain, Critical Care and Anaesthesia, “PCA”, of the Journal of Translational Medicine in which we welcome multidisciplinary contributions from colleagues interested in these areas of expertise.

## References

[1] Ashbaugh DG, Bigelow DB, Petty TL, Levine BE (1967). Acute respiratory distress in adults. Lancet.

[2] Bernard GR, Artigas A, Brigham KL, Carlet J, Falke K, Hudson L, Lamy M, LeGall JR, Morris A, Spragg R, Consensus Committee (1994). Report of the American-European Consensus Conference on acute respiratory distress syndrome: Definitions, mechanisms, relevant outcomes, and clinical trial coordination. J Crit Care.

[3] Thompson BT, Matthay MA (2013). “The Berlin sefinition of ARDS versus pathological evidence of diffuse alveolar damage”. Am J Respir Crit Care Med.

[4] Ranieri VM, Rubenfeld GD, Thompson BT, Ferguson ND, Caldwell E, Fan E, Camporota L, Slutsky AS (2012). Acute respiratory distress syndrome: the Berlin definition. JAMA.

[5] Fröhlich S, Murphy N, Boylan JF (2013). ARDS: progress unlikely with non-biological definition. Br J Anaesth.

[6] Bruehla S, Hardena RN, Galerb BS, Saltza S, Bertramc M, Backonjad M, Gaylese R, Rudinf N, Bhugrag MK, Stanton-Hicks M (1999). External validation of IASP diagnostic criteria for Complex Regional Pain Syndrome and proposed research diagnostic criteria. Pain.

[7] Norman Hardena R, Bruehlb S, Perezc RSGM, d’Birkleine F, Marinusd J, f’Maihofnerg C, Lubenowh T, Buvanendranh A, Mackeyi S, Graciosaa J, Mogilevskia M, Ramsdena C, Chontb M, Vatinej JJ (2010). Validation of proposed diagnostic criteria (the “Budapest Criteria”) for Complex Regional Pain Syndrome. Pain.

